# Tooth retention, health, and quality of life in older adults: a scoping review

**DOI:** 10.1186/s12903-022-02210-5

**Published:** 2022-05-18

**Authors:** Adejare Jay Atanda, Alicia A. Livinski, Steven D. London, Shahdokht Boroumand, Darien Weatherspoon, Timothy J. Iafolla, Bruce A. Dye

**Affiliations:** 1 National Institute of Dental and Craniofacial Research, National Institutes of Health, 31 Center Dr., Suite 5B55, Bethesda, MD USA; 2 National Library of Medicine, National Institutes of Health, Bethesda, MD USA; 3 National Institutes of Health Library, Office of Research Services, OD, National Institutes of Health, Bethesda, MD USA; 4 School of Dental Medicine, Stony Brook University, Stony Brook, NY USA; 5 School of Dentistry, University of Maryland, Baltimore, MD USA; 6 School of Dental Medicine, University of Colorado, Anschutz Medical Campus, Aurora, CO USA

**Keywords:** Functional dentition, Shortened dental arch, Tooth retention, Tooth loss, Quality of life

## Abstract

**Objective:**

This scoping review describes the relationship between tooth retention, health, and quality of life in older adults.

**Methods:**

Seven databases were searched for English language articles for subjects ≥ 65 y from 1981 to 2021. Exposure was tooth retention (≥ 20), and outcomes were general/systemic health and quality of life. Methodological quality was assessed using the Newcastle–Ottawa Scale and Cochrane Risk of Bias 2.0 tool.

**Results:**

140 articles were included, only four were randomized trials. Inter-rater agreement (κ) regarding study inclusion was 0.924. Most were assessed with low risk of bias (*n* = 103) and of good quality (*n* = 96). Most studies were conducted in Japan (*n* = 60) and Europe (*n* = 51) and only nine in the US. Tooth retention was referred to as “functional dentition” in 132 studies and “shortened dental arch” in 19 studies. Study outcomes were broadly synthesized as (1) cognitive decline/functional dependence, (2) health status/chronic diseases, (3) nutrition, and (4) quality of life.

**Discussion:**

There is a positive relationship between tooth retention, overall health, and quality of life. Older adults retaining ≥ 20 teeth are less likely to experience poorer health. Having < 20 teeth increases the likelihood for functional dependence and onset of disability, and may affect successful ageing. This review supports the general finding that the more teeth older adults retain as they age, the less likely they are to have adverse health outcomes. However, significant knowledge gaps remain which can limit decision-making affecting successful ageing for many older adults. This review highlights the need to consider, as an important marker of oral health and function, the retention of a functional minimum of a natural dentition, rather than a simple numeric score of missing teeth.

**Supplementary Information:**

The online version contains supplementary material available at 10.1186/s12903-022-02210-5.

## Introduction

The aging of the global population is a hallmark of the twenty-first century with average lifespans projected to reach 100 years in some countries [1]. The world’s population is ageing rapidly with a projected doubling of the population by 2050, with those aged 80 and older expected to triple by 2050. In the US, by 2034 older adults will surpass children in population size for the first time and by 2060, they will comprise one-quarter of the population [2, 3]. Many of these older adults have retained much of their natural dentition and this trend is expected to continue. As recently as 1970, complete tooth loss (edentulism), affected half of the US older adult population. Today, the edentulism rate is 17% and is projected to decrease to < 3% by 2050 [4–6]. Globally, edentulism has decreased by about half among all persons, regardless of age, from 1990 to 2010 [7].

Tooth retention is considered an important indicator of a population’s oral health and is monitored by several countries. For example, the *‘8020’ campaign* targeting elderly adults in Japan, which promotes the retention of ≥ 20 teeth by the time they reach the age of 80 years [8–10]. Research has shown an inverse correlation between masticatory function measured as number of remaining teeth and certain chronic systemic diseases [11–19]. Additional studies suggest cognitive and physical functioning may be poorer in edentulous older adults ≥ 65 years compared to their dentate counterparts [11, 20, 21]. There are also significant positive correlations between oral health status and overall/general health-related quality of life (HRQoL) [22–24], as well as the number of remaining teeth [25].

Quality of life (QoL) measures often include an objective and a subjective evaluation of life circumstances [26]. Fallowfield proposed psychological (e.g., depression), social (e.g., engagement in social and leisure activities), occupational (e.g., the ability to carry out paid or domestic work), and physical (e.g., pain, sleep, and mobility) core domains of QoL [27]. The definition of HRQoL varies, but the consensus identifies physical symptoms, perceptions of well-being and functional capacity as major dimensions [28]. Oral health contributes to QoL, and these dimensions are applied in dental research as number of *dental symptoms*, *perception of oral well-being*, and social and physical *oral functioning* [29–32].

Studying how tooth retention is related with health and QoL in aging populations is important because of the expected demographic shifts and tooth retention trends. Having a better understanding of the role tooth retention plays in health and wellbeing will help to inform how we can improve care for the increasing diversity and numbers of older adults whose oral health status and needs will be changing as well. Thus, our objective is to evaluate the literature using systematic methodology to ascertain the breadth of published information on tooth retention, health and QoL in older adults (≥ 65) using two common concepts, a functional dentition (FD) and a shortened dental arch (SDA), considered to be the minimal threshold of tooth retention needed to have a positive effect on well-being. This will help to identify gaps in knowledge and opportunities for research on the topics of tooth retention and health in older adults.

## Methods

This study is a scoping review with the purpose of identifying knowledge and conceptual gaps and describing a body of literature on the topic. A protocol was written a priori for the review using the Preferred Reporting Items for Systematic Reviews and Meta-Analyses extension for Scoping Reviews (PRISMA-ScR) [33]. We also used the PRISMA-ScR [33] to write this review.

Our primary research question was: What is the relationship between tooth retention defined as ≥ 20 teeth (characterized as either FD or SDA) on health and HRQoL in adults age ≥ 65? To guide this review, we established a set of a priori operational definitions:

*Functional dentition*: having ≥ 20 teeth regardless of position, location, or teeth type or ≥ 21 natural teeth [5, 34].

*Shortened dental arch*: is the presence of ≥ 20 teeth based on position and location on the dental arch dependent on author specific definitions focusing on the number of functional occlusal contacts in posterior teeth [35–37].

*HRQoL*: “An individual’s or group’s perceived physical and mental health over time” [38].

*Successful aging*: Having low probability of disease and disability, high cognitive and physical functioning, as well as productive activity and activity involving relations with others [39].

*Well-being*: Having positive outcomes that are meaningful including judgments of life satisfaction and feelings ranging from depression to joy. It’s what people think and feel about their lives, including quality of their relationships, their positive emotions and resilience, realization of their potential, and overall satisfaction with life [40].

### Eligibility criteria

To assist with identifying studies eligible for inclusion in this scoping review, we used the following PECO framework:

*Participants*: Adults ≥ 65, with no geographic or other demographic restrictions.

*Exposure*: Tooth retention, presence of ≥ 20/≥ 21 teeth, characterized as a FD/SDA.

*Comparators*: Included studies must compare outcomes in a group with FD/SDA and a group without FD/SDA.

*Outcomes:* Included studies were required to report on outcomes in one or more of four major categories: (1) Cognitive decline/functional dependence, (2) Health status/chronic diseases, (3) Nutrition, and/or (4) Quality of Life.

We limited the search to English language articles published from 1981 to 2021. In 1981, Kayser proposed the SDA concept [41], studies on FD followed later. Initial title/abstract (tiab) search included studies with participants ≥ 18, where exposure was tooth retention described as FD/SDA, and outcomes were general/systemic health and quality of life. Studies for full-text review were then limited to studies where participants age or age-groups were clearly defined as ≥ 65.

We included original observational research (cross-sectional, cohort, and case control studies) and interventional research (randomized and non-randomized controlled trials). Studies exclusively dealing with edentulous subjects with prostheses, in vitro studies, case series, case reports, non-peer reviewed studies, letters to the editor, editorials, commentaries, narrative syntheses, abstracts, personal communication as well as studies with both adults and children when data is not reported separately for adults ≥ 65 were excluded.

### Information sources and search

A research librarian (AAL) independently performed searches in December 2019 and September 2021 of 7 databases: Ageline (EBSCOhost), Cochrane Library Database of Systematic Reviews (Wiley and Sons), Cumulative Index of Nursing and Allied Health Literature (EBSCOhost), Embase (Elsevier), PubMed/MEDLINE (US National Library of Medicine), Scopus (Elsevier), and Web of Science: Core Collection (Clarivate Analytics). A combination of controlled vocabulary terms (CINAHL Subject Headings, EMTREE, Medical Subject Headings), key words, and phrases for each of the concepts of interest were adapted for each database (see Additional file 2). Search results were exported into EndNote X9 (Clarivate Analytics) for citation management and removal of duplicates, and into Covidence (Veritas Health Innovation) for tiab and full-text screening.

### Study selection, data collection, and risk of bias

Inter-rater reliability assessments were conducted between two main reviewers (AAA and SDL) and concordance was excellent before tiab and full-text screening (Kappa of 0.92 and 1.00 during tiab and full-text screening respectively). The main reviewers completed the initial tiab screen in February 2020 and the follow-up tiab in September 2021 when the review was updated, with another reviewer (SB) resolving disagreements from tiab screen using Covidence. Full-text articles were reviewed through September 2021 with two reviewers (SB, DW) serving as arbitrators.

Using Microsoft Excel, AAA and SDL independently extracted data into tables including author/year, objective, country, age, sample size, study design etc. Risk of bias and methodological quality was assessed using the Newcastle–Ottawa Scale (NOS) [42] for observational studies and the Cochrane Collaboration’s revised Risk of Bias 2.0 (RoB 2.0) for randomized trials [43] (Additional file 1: Table S3).

## Results

### Selection of sources

**Fig. 1:**
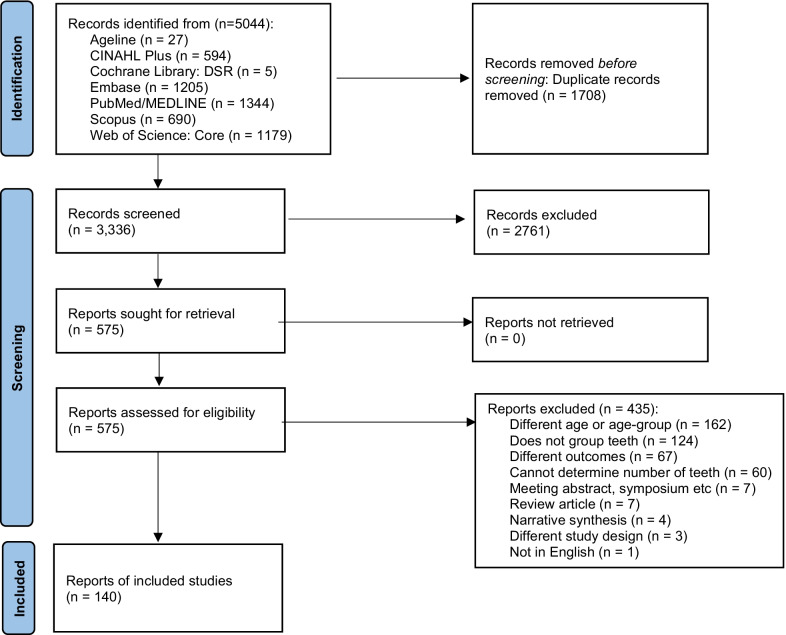
PRISMA flow diagram outlining search strategy and results along various steps

We retrieved 5044 citations after the database searches of which 1708 duplicates were removed resulting in 3336 unique citations for screening. After tiab screening, 575 studies were identified that met inclusion criteria. Following full-text screening, 435 were excluded (different age or age-groups, natural present teeth not grouped, different outcomes, inappropriate or incorrect study designs etc.) and 140 studies were included. See Fig. 1 for PRISMA flow diagram and full list of exclusion reasons.

### Article characteristics

General characteristics of the 140 included studies are provided in Additional file 1: Tables S1 and S2. All were observational studies except for four randomized controlled clinical trials (RCT). Two observational studies were from a retrospective cohort, 23 from a prospective cohort, and 111 were cross-sectional studies. The majority were conducted in Japan (*n* = 60) and Europe (*n* = 51). Most European studies originated from the UK (*n* = 11) with Brazil (*n* = 12) and South Korea (*n* = 9) representing most of the non-European studies. Nine studies were conducted in the US.

**Fig. 2:**
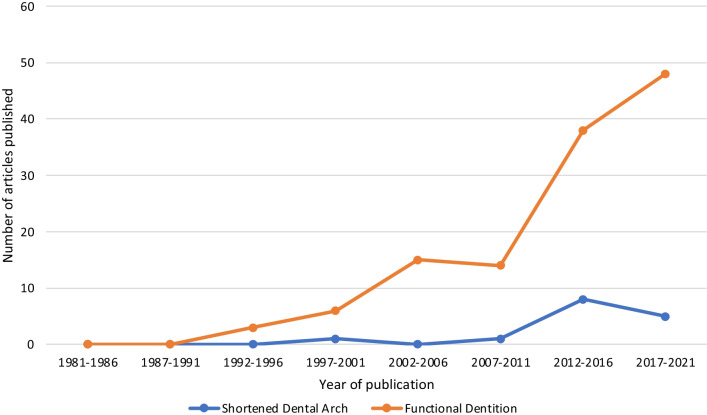
Frequency of publication by tooth retention concept and year

There were 130 studies defining tooth retention in terms of a FD: 78 studies defined it as retention of ≥ 20 teeth, 18 defined it as retention of ≥ 21 teeth, and 36 assessed a FD in other ways (example, in terms of missing teeth or some other category of teeth present) (Additional file 1: Table S1). Researchers in 14 studies defined tooth retention in terms of a SDA (Additional file 1: Table S2). Nine studies defined tooth retention in terms of both FD and SDA. Specific information by tooth retention concept for all studies are in Additional file 1: Tables S1 and S2. Most outcomes evaluated were cognitive decline/functional dependence (*n* = 52), QoL (*n* = 45) and health status/chronic diseases (*n* = 43) (Table 1). The frequency of the more recent studies published substantially favored the use of FD (Fig. 2).

**Table 1:** Number of articles (*n* = 140) in scoping review by outcome, study design, location, and “Tooth Retention Concept”

Medical or dental outcomes	Article study design by outcomes			Geographic location				Tooth retention concept		
	Observational study (research)	Interventional study (research)	*Total*	Japan	Europe	U.S.	Elsewhere	FD	SDA	FD + SDA
Cognitive decline/functional dependence	52	0	*52*	28	14	1	14	48	5	2
Quality of life	43	2	*45*	9	17	2	19	31	3	1
Health status/chronic diseases	41	2	*43*	17	12	4	11	38	5	3
Nutrition	18	2	*20*	6	8	2	5	15	6	3
Total	154	6	160	60	51	9	49	132	19	9

Columns don’t add up to 140—# of articles in this review. This is because of "double- or even triple-counting". For example, under the column, "geographic location", some studies were conducted in multiple countries, hence counted more than once. Some studies examined more than one outcome too for example. Same applies for all other columns

### Risk of bias

Using the NOS and RoB 2.0 tools to assess risk of bias and methodological quality, 103 studies had a low risk of bias and the methodological quality of evidence in 96 studies was good (Additional file 1: Table S3). Only nine of the 140 articles included in this review had a sample size less than 100, indicating the majority were likely of adequate size (Additional file 1: Tables S1 and S2).

### Synthesis of findings

#### Cognitive decline and functional dependence outcomes

The occurrence of severe cognitive impairment or lower mental status was associated with the presence of < 10 natural teeth in at least one dental arch (< 20 teeth total) [44, 45]. Having < 20 teeth is associated with lower hand-grip strength, leg extensor strength, isokinetic leg extensor power, and one-leg standing time, though the relationship tends to disappear after adjusting for confounders such as height, body weight, gender, smoking and alcohol, marital status, regular medical treatment, and regular exercise [46]. Activities of daily living/instrumental activities of daily living (ADL/IADL) problems were associated with < 20 teeth and loss of a FD was associated with severe locomotion impairments and difficulties with ADL/IADL [47–57]. In addition, having < 20 teeth was associated with functional dependence and was associated with onset of disability, declines in higher-level functional and mortality [47, 49, 53, 54, 58, 59]. Wearing dental prostheses mediated these effects (e.g., subjects having 19 or fewer teeth but *not* wearing dentures had a significantly increased risk for incident falls compared with those having FD, though wearing dentures alleviated this risk) [60].

Persons with FD were more active in leisure sports suggesting that the number of remaining teeth was associated with functional capacity, physical ability, and physical activity in elderly persons [50, 61–63]. A Higher Mini-Mental State Examination ‘MMSE’ scores (cognitive decline) were associated with FD compared to < 20 teeth after adjustment for demographics in a relationship also moderated/mediated by prostheses type [55, 56, 64–69]. Results on FD and psychological distress and depression are mixed. Self-rated poor oral health increased for individuals who had symptoms of psychological distress and < 20 teeth [70].

#### Health status/chronic diseases outcomes

Older adults with < 20 teeth present had 2.5 times greater risk of cardiovascular disease, a relationship mediated by weight and BMI, suggesting that the number of missing teeth affects the quality of diet [71]. One study reported a relationship between tooth loss and chronic kidney disease, especially among older women [72]. Older adults with < 20 teeth had significantly increased risk of metabolic syndrome, higher serum lipid peroxide, and glycemic status [73–75]. However, the relationship was reversed in a study population of Medicaid patients for glycemic status [73].

Favorable lifestyles such as “no smoking” and “no alcohol drinking” show a strong association with > 20 teeth with the inverse being true for those with < 20 teeth or lacking a FD [76–78]. Similar relationships were reported where smoking and drinking status were used as confounders, though these results were mixed when the health outcome was hypertension [79–81]. Older Brazilian adults with ≥ 20 teeth had a lower chance of being frail than edentulous individuals, and elderly individuals with a need for dental prostheses were significantly more likely to be prefrail and frail [82]. Similar results were reported in Chinese, Japanese, and Danish cohorts though in a British cohort, this association was not significant [83–87]. In those ≥ 85 y, those with FD had a longer life expectancy and demonstrated successful ageing when compared with the edentulous participants [88]. Individuals with FD had more skeletal muscle mass than those with ≤ 20 teeth [89].

#### Nutrition outcomes

Older adults with a compromised FD (≤ 20 teeth) had higher odds of inadequate calorie intake, lower Healthy Eating Index (HEI) scores, and BMI outside of the normal range (obesity or being underweight) [52, 90–95]. In randomized, controlled clinical trials, conventional and functionally oriented tooth replacement strategies both showed significant improvement in mini-nutritional assessment scores though hematological markers did not provide a clear picture of improvement in either group [96, 97]. In general, people with FD were significantly more likely to be able to chew all foods, have a normal BMI, and consume more nutrients [98–100].

#### Quality of life outcomes

Sáez-Prado and colleagues showed a relationship between summary HRQoL variables in EuroQol- 5 Dimension (EQ-5D), visual analogue scale (VAS) and Oral Health Impact Profile (OHIP-14) scores [101]. Using the EQ-5D, those with ≤ 20 teeth were significantly less likely to have a good score in any of its five dimensions [102]. Dissatisfaction with daily life and an unfavorable face-scale score (negative mood) are more prevalent in those with < 20 teeth. However there was no consistent difference in prevalence of poor QoL status when comparing four groups defined by remaining numbers of teeth by regression analysis [103] though Wu et al. report that higher number of occluding tooth pairs were associated with better oral health-related quality of life (OHRQoL) (*p* < 0.001) [104]. Having ≤ 20 teeth mediates the association between living alone, measures of social relations, and coronal caries [105–107].

In general, social participation measures (travel, participation in leisure sports, cultural activity, neighborhood associations, or hobby clubs), were related to having ≥ 20 teeth [61, 106]. Using the Short Form Health Survey (SF-36), those with ≥ 20 teeth had higher physical component scores than did those with ≤ 19 teeth [26]. A SDA resulted in better or smaller OHIP-14 scores i.e. better OHRQoL compared to a removable partial denture group in one RCT [108].

## Discussion

This scoping review describes the current state of knowledge regarding tooth retention, general/systemic health, and quality of life in older adults. Oral disease is a universal problem, but because it is rarely life-threatening, its prevention or treatment is often a low priority for health policy makers [31]. For instance, almost half of the world’s population is affected by an oral condition, accounting for 0.6% of all disability adjusted life-years (DALYs) [109]. DALYs increased 20.8% from 1990 to 2010 but mainly due to population growth and aging [109]. Continuing trends of improving tooth retention and increasing chronic disease co-morbidities, common in people as they age past 65 years, point towards future unmet dental and other healthcare needs when placed in the context of health professional shortages especially in the field of geriatric dentistry.

Of the 140 articles included in this review, most were cross-sectional, published in the last decade, described tooth retention in terms of FD, and examined cognitive decline, functional dependence, and general health/chronic disease outcomes in Japanese or European populations.

Given the broad nature of our research question, this scoping review is helpful for identifying areas where more work is needed. For example, within the cognitive decline and functional dependence domain, most research examined ADL/IADL. However, there appears to be a lack of research ascertaining dementia and other cognitive screening tools and their relationship with tooth retention. Such tools are increasingly being recommended for use in primary care [110]. Two Japanese studies in this review investigated differences between *8020* and non-*8020* elderly [8, 9]. In general, *8020* achievers had good ADL/IADL levels and better satisfaction with life. Tooth loss is associated with onset of disability and mortality in old age and may be an early indicator of accelerated aging [49]. Maintenance of a FD is preventive against late-life cognitive declines in the aged population and associated with functional independence in nursing home residents [53, 55, 56]. Antunes and colleagues noted the complex interplay of factors resulting in a bidirectional relationship between dentition status and onset of functional disability [57]. For example, tooth brushing was reported to at least partially negate the increased risk of incident functional disability associated with having fewer remaining teeth [58], whereas Yamamoto and colleagues reported that in subjects with 19 or fewer teeth, the risk of falls was not significantly elevated as long as they wore dentures [60].

The relationship between tooth loss, numbers of remaining teeth, cognitive function, impairment or dementia, the direction of causality of the relationship between cognitive impairment and having poor oral health is poorly elucidated. Peres and colleagues reported a bidirectional association with effects particularly pronounced in older adults [44]. In Japan, where a sizable number of studies were conducted, dementia is a major cause of disruption of a healthy life expectancy. Kato et al. and Yoo et al. reported an association between number of teeth and cognitive function in community dwelling individuals [111]. Others report the use of artificial teeth, dentures, posterior teeth occlusion, socioeconomic status, educational level, smoking status, and systemic diseases, also influence this relationship [44, 69]. At least one study compared associations between dentate groups having FD, ≤ 19 teeth with dentures, and ≤ 19 teeth without dentures [69]. Participants with few teeth and without dentures had a nearly two-fold increased risk for dementia onset compared with those with 20 teeth or more [69].

Laurence Frank in 1946 presented two conflicting views of aging: one, an involutionary, largely pathological process; while the other describes a process of biological transformation [112]. The concept of successful ageing today is interconnected with QoL, a multidimensional construct that relates to the satisfaction of individual needs for growth, well-being, self-esteem, freedom and the pleasures of meaningful relationships and work [38, 40, 113, 114]. Based on the WHO model of health, Locker’s conceptualization of the impact of oral disease posits five consequences of oral disease: impairment, functional limitation, pain/discomfort, disability, and handicap [32, 115]. Implicit to Locker’s model is the assumption there is a relationship between poor oral health and impaired QoL. The medical model emphasizes the biological and pathological progression of oral health diseases and its irreversible endpoint, tooth loss; on the other hand, it uses measurable clinical indicators such as decayed, missing and filled teeth (DMFT), or numbers of remaining teeth as we do in this review.

Globally, 7 of the top 10 causes of mortality are ascribed to non-communicable diseases (NCDs) led by heart disease, stroke, and chronic obstructive pulmonary disease (COPD) [116]. In the US, non-communicable diseases (NCDs) account for 89% of all deaths and have surpassed infectious diseases as the main cause of death [117]. The top 5 NCDs are ischemic heart disease, Alzheimer’s, lung cancer, stroke, and COPD [117]. Yet, the paucity of longitudinal studies incorporating measures of tooth retention significantly limits our understanding of the relationship between tooth retention and chronic diseases. Within the nutrition domain, we note that studies generally focused on chewing ability and numbers of different types of foods/nutrients consumed rather using diet quality indices that are more appropriate for epidemiological studies. Within the QoL domain, few studies examined the relationship of tooth retention with social participation measures, which may be a significant oversight given the important esthetic and social considerations associated with tooth retention.

Only 20 articles described nutritional outcomes, suggesting the need for more, appropriately designed studies, conducted in the US and elsewhere to address this research gap. Within the nutritional outcome’s literature for instance, we also note that few used objective, diet quality measures of nutrition status like HEI, FDSK-11 or Diet Variety Score. Given that the US is becoming more diverse and aging rapidly, the lack of high-quality studies to inform evidence-based decision-making to improve oral health and related outcomes for older adults is a concern. Having > 20 teeth into old age played an important role in having a healthy diet rich in fruits and vegetables, a satisfactory nutritional status, and an acceptable BMI [98, 118, 119]. In the oldest old (80+ years), the presence of natural teeth, plus well-fitting dentures were associated with higher and more varied nutrient intakes and greater dietary quality [119]. The status of dentition should be a major consideration in nutritional counseling and assessment of older adults because tooth loss can affect ability to chew resulting in a lack of adequate nutrition intake [93].

Although we focused on FD/SDA and not edentulism, some of these articles reported a separate set of findings on edentulism and tooth loss or examined how having a denture or oral health behaviors like toothbrushing altered the relationship between tooth retention and the main outcomes considered in this review. For instance, severe cognitive impairment or a reduced mental status was associated with being edentulous [44, 45]. Edentulous subjects also had significantly increased risk of metabolic syndrome, higher serum lipid peroxide, and glycemic status [73–75], and Yamamoto and colleagues reported an association between edentulism and incident depressive symptoms [120]. While it may not be surprising that complete edentulism is associated with greater odds of self-rated “fair/poor” health in older diabetics, who also experienced additional physically unhealthy days in the past 30 days, it is also notable that the loss of *any* permanent teeth was also associated with these findings [121]. In addition, determinants of oral health behaviors were independently associated with edentulism [122], with tooth loss associated with self-limited food choices for example [12, 98, 99].

We encountered limitations common to reviews of the literature, for example difficulty in accessing literature not published in English, reporting on publication bias, and dependency on the rigor of methodological reporting in the included studies. In terms of strengths, most reviewed studies had a sample size > 100, had low risk of bias (*n* = 96) and were of good methodological quality (*n* = 103), as assessed using the NOS and RoB tools. This current review supersedes existing work in the following ways: (1) a focus solely on general/systemic health outcomes in older adults ≥ 65 rather than dental outcomes, (2) it uses common concepts that provide a threshold of tooth loss with minimal impact on well-being (FD/SDA), and (3) uses a longer timeframe for electronic database search. Although this review focused on numbers of remaining teeth, several studies described tooth retention in terms of tooth loss highlighting the need for a standardization of language that more appropriately describes the natural tooth retention trends we now see. This aligns with global efforts promoting retention of teeth as a healthcare policy goal.

## Conclusion

The high global prevalence of oral disorders [123] and the new effort underway by the WHO to develop a global oral health strategy (resolution WHA74.5) to improve oral health and wellbeing that is aligned with NCD prevention strategies and universal health coverage [124] clearly points to the need for substantially more research to inform decision-making. As we move towards more integrated care models, cooperation among all health care providers will facilitate improved oral health among older adults, especially for those with multiple chronic conditions [50, 71]. With a “greying” global population, understanding how tooth retention patterns can affect successful aging among older adults, including where they live and work, is an important consideration for guiding evidence-based practice, including improving standards of care for those who are dependent on others for daily living, with the overall goal of promoting oral health and facilitating quality of life. Despite some uncertainty determining the direction of the cause and effect between tooth loss and overall health, insights from new research appear promising to facilitate clinical decision-making and caregiving, improve the framing of communication and health education strategies, and provide a guide for health policy makers who want to address the future oral health needs of older adults.

## Supplementary Information

**Additional file 1:** Supplementary Tables 1-3 (Outcomes by Tooth Retention Concept, Risk of Bias and Quality/Strength of Cumulative Evidence).

**Additional file 2:** Supplementary Information. Search Strategy for Seven Databases.

## Data Availability

All data generated or analyzed during this study are included in this published article and its Additional files 1 and 2.

## Abbreviations

QoL: Quality of life
HRQoL: Health-related quality of life
FD: Functional dentition
SDA: Shortened dental arch
PRISMA-ScR: Preferred reporting items for systematic reviews and meta-analyses extension for scoping reviews
PECO: Participants, exposure, comparators, outcomes
NOS: Newcastle–Ottawa Scale
RoB2: Cochrane collaboration’s revised risk of bias tool 2.0
OHRQoL: Oral health-related quality of life
