# Supplementary material for: Tooth retention, health, and quality of life in older adults: a scoping review
Source: BMC Oral Health. 2022 May 18;22:185. doi: 10.1186/s12903-022-02210-5 (PMC9118621; doi:10.1186/s12903-022-02210-5)
Supplement: Supplementary file 2 — Additional file 2: Supplementary Information. Search Strategy for Seven Databases. [file 12903_2022_2210_MOESM2_ESM.docx]

**Supplementary Information. Search Strategy for Seven Databases.**

Database: PubMed/MEDLINE

Vendor: National Library of Medicine

Date: December 19, 2019

Limits: Language: English; Publication date: 1/1/1981 – 12/31/2020; Subset: MEDLINE[sb]

Notes: Use Advanced Search and apply the MEDLINE subset. Apply limits for publication date and language from the search results page using the filters on the left-side.

(geriatric[tiab] OR geriatrics[tiab] OR elderly[tiab] OR senior[tiab] OR seniors[tiab] OR elder[tiab] OR elders[tiab] OR aging[tiab] OR ageing[tiab] OR "older adult"[tiab] OR "older adults"[tiab] OR “older patient”[tiab] OR "older patients"[tiab] OR “aging patient”[tiab] OR “aging patients”[tiab] OR “older people”[tiab] OR “older person”[tiab] OR “older persons”[tiab] OR "aged 65 years"[tiab] OR Aged[mesh] OR geriatrics[mesh] OR frail elderly[mesh] OR "Aged, 80 and over"[Mesh]) AND ("shortened dental arch"[tw] OR "shortened dental arches"[tw] OR "tooth retention"[tiab] OR "teeth retention"[tiab] OR "tooth loss"[tiab] OR "teeth loss"[tiab] OR "lacking teeth"[tiab] OR "Tooth Loss"[Mesh] OR "Dental Arch"[Mesh] OR "Dentition"[Mesh] OR "functional dentition"[tiab] OR "dental loss"[tiab] OR "tooth arch"[tiab] OR "tooth arches"[tiab]) AND (“oral health quality of life”[tw] OR OHQOL[tw] OR OHRQOL[tw] OR OHRQL[tw] OR “health related quality of life”[tw] OR HRQOL[tw] OR HQOL[tw] OR HRQL[tw] OR “quality of life”[tiab] OR “life quality”[tiab] OR “quality of living”[tiab] OR “patient satisfaction”[tiab] OR “successful aging”[tiab] OR “successful ageing”[tiab] OR “healthy aging”[tiab] OR “healthy ageing”[tiab] OR “robust aging”[tiab] OR “active aging”[tiab] OR “active ageing”[tiab] OR “positive aging”[tiab] OR “positive ageing”[tiab] OR “ageing well”[tiab] OR “aging well”[tiab] OR “optimal aging”[tiab] OR “optimal ageing”[tiab] OR “productive aging”[tiab] OR “productive ageing”[tiab] OR “effective aging”[tiab] OR “masticatory function”[tiab] OR “masticatory functioning”[tiab] OR “masticatory functions”[tiab] OR mastication[tiab] OR “well-being”[tiab] OR wellbeing[tiab] OR wellness[tiab] OR “aesthetic appearance”[tiab] OR “aesthetic appearances”[tiab] OR “physical appearance”[tiab] OR “physical appearances”[tiab] OR “cognitive function”[tiab] OR “cognitive functions”[tiab] OR “cognitive functioning”[tiab] OR “physical function”[tiab] OR “physical functions”[tiab] OR “physical functioning”[tiab] OR “nutritional status”[tiab] OR “nutrition status”[tiab] OR “health outcome”[tiab] OR “health outcomes”[tiab] OR “health status”[tiab] OR "Quality of Life"[Mesh] OR "Cognition"[Mesh] OR "Mastication"[Mesh] OR "Patient Satisfaction"[Mesh] OR "Healthy Aging"[Mesh] OR "Cognitive Aging"[Mesh] OR "Health Status"[Mesh] OR "Nutritional Status"[Mesh] OR "Physical Appearance, Body"[Mesh] OR WHOQOL[tw] OR “WHO QOL”[tw])

AND (("1981/01/01"[PDAT] : "3000/12/31"[PDAT]) AND (Spanish[lang] OR English[lang])) AND medline[sb])

Database: Embase

Vendor: Elsevier

Date: December 27, 2019

Limits: Language: English, Spanish; Publication date: 1981-2020; Source: Embase, Embase Classic

Note: Use Advanced Search and apply the language, publication date and source limits at the Advanced Search page under the search box.

(geriatric:ti,ab OR geriatrics:ti,ab OR elderly:ti,ab OR senior:ti,ab OR seniors:ti,ab OR elder:ti,ab OR elders:ti,ab OR aging:ti,ab OR ageing:ti,ab OR "older adult":ti,ab OR "older adults":ti,ab OR “older patient”:ti,ab OR "older patients":ti,ab OR “aging patient”:ti,ab OR “aging patients”:ti,ab OR “older person”:ti,ab OR “older persons”:ti,ab OR “older people”:ti,ab OR "aged 65 years":ti,ab OR 'aged'/de OR 'very elderly'/exp OR 'frail elderly'/exp OR 'aged hospital patient'/exp OR 'institutionalized elderly'/exp) AND (“shortened dental arch”:ti,ab OR “shortened dental arches”:ti,ab OR “tooth retention”:ti,ab OR “teeth retention”:ti,ab OR “tooth loss”:ti,ab OR “teeth loss”:ti,ab OR “lacking teeth”:ti,ab OR “dental arch”:ti,ab OR “functional dentition”:ti,ab OR “dental loss”:ti,ab OR “tooth arch”:ti,ab OR “tooth arches”:ti,ab OR 'tooth arch'/exp OR 'dentition'/exp) AND (“oral health quality of life”:ti,ab OR OHQOL:ti,ab OR OHRQOL:ti,ab OR OHRQL:ti,ab OR “health related quality of life”:ti,ab OR HRQOL:ti,ab OR HQOL:ti,ab OR “quality of life”:ti,ab OR “life quality”:ti,ab OR “quality of living”:ti,ab OR HRQL:ti,ab OR “patient satisfaction”:ti,ab OR “successful aging”:ti,ab OR “successful ageing”:ti,ab OR “healthy aging”:ti,ab OR “healthy ageing”:ti,ab OR “robust aging”:ti,ab OR “active aging”:ti,ab OR “active ageing”:ti,ab OR “positive aging”:ti,ab OR “positive ageing”:ti,ab OR “ageing well”:ti,ab OR “aging well”:ti,ab OR “optimal aging”:ti,ab OR “optimal ageing”:ti,ab OR “productive aging”:ti,ab OR “productive ageing”:ti,ab OR “effective aging”:ti,ab OR mastication:ti,ab OR “masticatory function”:ti,ab OR “masticatory functioning”:ti,ab OR “masticatory functions”:ti,ab OR “well-being”:ti,ab OR wellbeing:ti,ab OR wellness:ti,ab OR “aesthetic appearance”:ti,ab OR “aesthetic appearances”:ti,ab OR “physical appearance”:ti,ab OR “physical appearances”:ti,ab OR “cognitive function”:ti,ab OR “cognitive functions”:ti,ab OR “cognitive functioning”:ti,ab OR “physical function”:ti,ab OR “physical functions”:ti,ab OR “physical functioning”:ti,ab OR “nutritional status”:ti,ab OR “nutrition status”:ti,ab OR “health outcome”:ti,ab OR “health outcomes”:ti,ab OR “health status”:ti,ab OR WHOQOL:ti,ab OR “WHO QOL”:ti,ab OR “healthy days measures”:ti,ab OR 'quality of life'/exp OR 'healthy aging'/exp OR 'health outcomes'/exp OR 'health status'/exp OR 'physical appearance'/exp OR 'wellbeing'/exp OR 'cognitive aging'/exp OR 'physical function'/exp OR 'physical functioning'/exp OR 'patient satisfaction'/exp OR 'nutritional status'/exp OR 'mastication'/exp)

AND ([english]/lim OR [spanish]/lim) AND [1981-2020]/py AND ([embase]/lim OR [embase classic]/lim)

Database: Scopus

Vendor: Elsevier

Date: December 19, 2019

Limits: Language: English, Spanish; Publication date: 1981-2020;

Note: Use Advanced Search and apply the language and publication date limits using the Refine Results on the search results page.

Title-Abs-Key( (geriatric OR geriatrics OR elderly OR senior OR seniors OR elder OR elders OR aging OR ageing OR {older adult} OR {older adults} OR {older patient} OR {older patients} OR {aging patient} OR {aging patients} OR {older person} OR {older persons} OR {older people} OR {aged 65 years} OR {aged hospital patient}) AND ({shortened dental arch} OR {shortened dental arches} OR {tooth retention} OR {teeth retention} OR {tooth loss} OR {teeth loss} OR {lacking teeth} OR {dental arch} OR {functional dentition} OR {dental loss} OR {tooth arch} OR {tooth arches}) AND ({oral health quality of life} OR OHQOL OR OHRQOL OR OHRQL OR {health related quality of life} OR HRQOL OR HQOL OR HRQL OR {quality of life} OR {life quality} OR {quality of living} OR {patient satisfaction} OR {successful aging} OR {successful ageing} OR {healthy aging} OR {healthy ageing} OR {robust aging} OR {active aging} OR {active ageing} OR {positive aging} OR {positive ageing} OR {ageing well} OR {aging well} OR {optimal aging} OR {optimal ageing} OR {productive aging} OR {productive ageing} OR {effective aging} OR mastication OR {masticatory function} OR {masticatory functioning} OR {masticatory functions} OR {well-being} OR wellbeing OR wellness OR {aesthetic appearance} OR {aesthetic appearances} OR {physical appearance} OR {physical appearances} OR {cognitive function} OR {cognitive functions} OR {cognitive functioning} OR {physical function} OR {physical functions} OR {physical functioning} OR {nutritional status} OR {nutrition status} OR {health outcome} OR {health outcomes} OR {health status} OR WHOQOL OR {WHO QOL} OR {healthy days measures} ))

Database: Web of Science: Core Collection

Vendor: Clarivate Analytics

Date: December 19, 2019

Limits: Language: English, Spanish; Publication date: 1981-2020; Field: Topic search

Note: Use Advanced search and apply the date and language limits.

TS=( (geriatric OR geriatrics OR elderly OR senior OR seniors OR elder OR elders OR aging OR ageing OR "older adult" OR "older adults" OR "older patient" OR "older patients" OR "aging patient" OR "aging patients" OR "older person" OR "older persons" OR "older people" OR "aged 65 years" OR "aged hospital patient") AND ("shortened dental arch" OR "shortened dental arches" OR "tooth retention" OR "teeth retention" OR "tooth loss" OR "teeth loss" OR "lacking teeth" OR "dental arch" OR "functional dentition" OR "dental loss" OR "tooth arch" OR "tooth arches") AND ("oral health quality of life" OR OHQOL OR OHRQOL OR OHRQL OR "health related quality of life" OR HRQOL OR HQOL OR HRQL OR "quality of life" OR "life quality" OR "quality of living" OR "patient satisfaction" OR "successful aging" OR "successful ageing" OR "healthy aging" OR "healthy ageing" OR "robust aging" OR "active aging" OR "active ageing" OR "positive aging" OR "positive ageing" OR "ageing well" OR "aging well" OR "optimal aging" OR "optimal ageing" OR "productive aging" OR "productive ageing" OR "effective aging" OR mastication OR "masticatory function" OR "masticatory functioning" OR "masticatory functions" OR "well-being" OR wellbeing OR wellness OR "aesthetic appearance" OR "aesthetic appearances" OR "physical appearance" OR "physical appearances" OR "cognitive function" OR "cognitive functions" OR "cognitive functioning" OR "physical function" OR "physical functions" OR "physical functioning" OR "nutritional status" OR "nutrition status" OR "health outcome" OR "health outcomes" OR "health status" OR WHOQOL OR "WHO QOL" OR "healthy days measures") )

Database: CINAHL Plus

Vendor: EBSCOhost

Date: December 19, 2019

Limits: Language: English, Spanish; Publication date: 1981-2020; Fields: Title, Abstract, Exact Subject Heading

Note: Use Advanced Search and use Search History to combine search sets together. Use Edit from the Search History page to apply limits to the search.

Title: (geriatric OR geriatrics OR elderly OR senior OR seniors OR elder OR elders OR aging OR ageing OR "older adult" OR "older adults" OR “older patient” OR "older patients" OR “aging patient” OR “aging patients” OR “older people” OR “older person” OR “older persons” OR "aged 65 years")

OR Abstract: (geriatric OR geriatrics OR elderly OR senior OR seniors OR elder OR elders OR aging OR ageing OR "older adult" OR "older adults" OR “older patient” OR "older patients" OR “aging patient” OR “aging patients” OR “older people” OR “older person” OR “older persons” OR "aged 65 years")

OR Exact Subject Heading: (MH "Aged+") OR (MH "Aged, 80 and Over") OR (MH "Geriatrics")

AND

Title: ("shortened dental arch" OR "shortened dental arches" OR "tooth retention" OR "teeth retention" OR "tooth loss" OR "teeth loss" OR "lacking teeth" OR "functional dentition" OR "dental loss" OR "tooth arch" OR "tooth arches")

OR Abstract: ("shortened dental arch" OR "shortened dental arches" OR "tooth retention" OR "teeth retention" OR "tooth loss" OR "teeth loss" OR "lacking teeth" OR "functional dentition" OR "dental loss" OR "tooth arch" OR "tooth arches")

OR Exact Subject Heading: (MH "Tooth Loss") OR (MH "Dentition+") OR (MH "Dental Arch")

AND

Title: (“oral health quality of life” OR OHQOL OR OHRQOL OR OHRQL OR “health related quality of life” OR HRQOL OR HQOL OR HRQL OR “quality of life” OR “life quality” OR “quality of living” OR “patient satisfaction” OR “successful aging” OR “successful ageing” OR “healthy aging” OR “healthy ageing” OR “robust aging” OR “active aging” OR “active ageing” OR “positive aging” OR “positive ageing” OR “ageing well” OR “aging well” OR “optimal aging” OR “optimal ageing” OR “productive aging” OR “productive ageing” OR “effective aging” OR “masticatory function” OR “masticatory functioning” OR “masticatory functions” OR mastication OR “well-being” OR wellbeing OR wellness OR “aesthetic appearance” OR “aesthetic appearances” OR “physical appearance” OR “physical appearances” OR “cognitive function” OR “cognitive functions” OR “cognitive functioning” OR “physical function” OR “physical functions” OR “physical functioning” OR “nutritional status” OR “nutrition status” OR “health outcome” OR “health outcomes” OR “health status” OR WHOQOL OR “WHO QOL” OR "healthy days measures")

OR Abstract: (“oral health quality of life” OR OHQOL OR OHRQOL OR OHRQL OR “health related quality of life” OR HRQOL OR HQOL OR HRQL OR “quality of life” OR “life quality” OR “quality of living” OR “patient satisfaction” OR “successful aging” OR “successful ageing” OR “healthy aging” OR “healthy ageing” OR “robust aging” OR “active aging” OR “active ageing” OR “positive aging” OR “positive ageing” OR “ageing well” OR “aging well” OR “optimal aging” OR “optimal ageing” OR “productive aging” OR “productive ageing” OR “effective aging” OR “masticatory function” OR “masticatory functioning” OR “masticatory functions” OR mastication OR “well-being” OR wellbeing OR wellness OR “aesthetic appearance” OR “aesthetic appearances” OR “physical appearance” OR “physical appearances” OR “cognitive function” OR “cognitive functions” OR “cognitive functioning” OR “physical function” OR “physical functions” OR “physical functioning” OR “nutritional status” OR “nutrition status” OR “health outcome” OR “health outcomes” OR “health status” OR WHOQOL OR “WHO QOL” OR "healthy days measures")

OR Exact Subject Heading: (MH "Quality of Life") OR (MH "Cognition") OR (MH "Mastication") OR (MH "Personal Appearance+") OR (MH "Patient Satisfaction") OR (MH "Healthy Aging") OR (MH "Cognitive Aging") OR (MH "Health Status") OR (MH "Nutritional Status")

Database: Ageline

Vendor: EBSCOhost

Date: December 19, 2019

Limits: Publication date: 1981-2020; Field: Title, Abstract, Ageline Thesaurus Subject Headings

Note: Use Advanced Search and Search History to combine sets together and apply limits.

#1 TI (geriatric OR geriatrics OR elderly OR senior OR seniors OR elder OR elders OR aging OR ageing OR "older adult" OR "older adults" OR “older patient” OR "older patients" OR “aging patient” OR “aging patients” OR “older people” OR “older person” OR “older persons” OR "aged 65 years")

#2 AB (geriatric OR geriatrics OR elderly OR senior OR seniors OR elder OR elders OR aging OR ageing OR "older adult" OR "older adults" OR “older patient” OR "older patients" OR “aging patient” OR “aging patients” OR “older people” OR “older person” OR “older persons” OR "aged 65 years")

#3 DE "Older Adults" OR DE "65 " OR DE "70 " OR DE "75 " OR DE "80 " OR DE "85 " OR DE "90 " OR DE "95 " OR DE "Centenarians" OR DE "Old Old" OR DE "Geriatrics" OR DE "Aging"

#4 #1 OR #2 OR #3

#5 TI ("shortened dental arch" OR "shortened dental arches" OR "tooth retention" OR "teeth retention" OR "tooth loss" OR "teeth loss" OR "lacking teeth" OR "functional dentition" OR "dental loss" OR "tooth arch" OR "tooth arches")

#6 AB ("shortened dental arch" OR "shortened dental arches" OR "tooth retention" OR "teeth retention" OR "tooth loss" OR "teeth loss" OR "lacking teeth" OR "functional dentition" OR "dental loss" OR "tooth arch" OR "tooth arches")

#7 #5 OR #6

#8 TI (“oral health quality of life” OR OHQOL OR OHRQOL OR OHRQL OR “health related quality of life” OR HRQOL OR HQOL OR HRQL OR “quality of life” OR “life quality” OR “quality of living” OR “patient satisfaction” OR “successful aging” OR “successful ageing” OR “healthy aging” OR “healthy ageing” OR “robust aging” OR “active aging” OR “active ageing” OR “positive aging” OR “positive ageing” OR “ageing well” OR “aging well” OR “optimal aging” OR “optimal ageing” OR “productive aging” OR “productive ageing” OR “effective aging” OR “masticatory function” OR “masticatory functioning” OR “masticatory functions” OR mastication OR “well-being” OR wellbeing OR wellness OR “aesthetic appearance” OR “aesthetic appearances” OR “physical appearance” OR “physical appearances” OR “cognitive function” OR “cognitive functions” OR “cognitive functioning” OR “physical function” OR “physical functions” OR “physical functioning” OR “nutritional status” OR “nutrition status” OR “health outcome” OR “health outcomes” OR “health status” OR WHOQOL OR “WHO QOL” OR "healthy days measures")

#9 AB (“oral health quality of life” OR OHQOL OR OHRQOL OR OHRQL OR “health related quality of life” OR HRQOL OR HQOL OR HRQL OR “quality of life” OR “life quality” OR “quality of living” OR “patient satisfaction” OR “successful aging” OR “successful ageing” OR “healthy aging” OR “healthy ageing” OR “robust aging” OR “active aging” OR “active ageing” OR “positive aging” OR “positive ageing” OR “ageing well” OR “aging well” OR “optimal aging” OR “optimal ageing” OR “productive aging” OR “productive ageing” OR “effective aging” OR “masticatory function” OR “masticatory functioning” OR “masticatory functions” OR mastication OR “well-being” OR wellbeing OR wellness OR “aesthetic appearance” OR “aesthetic appearances” OR “physical appearance” OR “physical appearances” OR “cognitive function” OR “cognitive functions” OR “cognitive functioning” OR “physical function” OR “physical functions” OR “physical functioning” OR “nutritional status” OR “nutrition status” OR “health outcome” OR “health outcomes” OR “health status” OR WHOQOL OR “WHO QOL” OR "healthy days measures")

#10 DE "Successful Aging" OR DE "Healthy Aging" OR DE "Quality of Life" OR DE "Personal Appearance" OR DE "Cognition" OR DE "Nutritional Status" OR DE "Health Status"

#11 #8 OR #9 OR #10

#12 #4 AND #7 AND #11

Database: Cochrane Library: Database of Systematic Reviews

Vendor: Wiley & Sons

Date: December 19, 2019

Limits: Publication date: 1981-2020; Field: Title, Abstract, Keywords, MeSH; Content Type: Cochrane Reviews

Notes: Use search manager to apply the limits for publication date, field, and content type.

#1 (geriatric OR geriatrics OR elderly OR senior OR seniors OR elder OR elders OR aging OR ageing OR "older adult" OR "older adults" OR “older patient” OR "older patients" OR “aging patient” OR “aging patients” OR “older people” OR “older person” OR “older persons” OR "aged 65 years"):ti,ab,kw

#2 [mh Aged] OR [mh geriatrics] OR [mh “frail elderly”] OR [mh “Aged, 80 and over”]

#3 #1 OR #2

#4 ("shortened dental arch" OR "shortened dental arches" OR "tooth retention" OR "teeth retention" OR "tooth loss" OR "teeth loss" OR "lacking teeth" OR "functional dentition" OR "dental loss" OR "tooth arch" OR "tooth arches"):ti,ab,kw

#5 [mh "Tooth Loss"] OR [mh “Dental Arch”] OR [mh Dentition]

#6 #4 OR #5

#7 (“oral health quality of life” OR OHQOL OR OHRQOL OR OHRQL OR “health related quality of life” OR HRQOL OR HQOL OR HRQL OR “quality of life” OR “life quality” OR “quality of living” OR “patient satisfaction” OR “successful aging” OR “successful ageing” OR “healthy aging” OR “healthy ageing” OR “robust aging” OR “active aging” OR “active ageing” OR “positive aging” OR “positive ageing” OR “ageing well” OR “aging well” OR “optimal aging” OR “optimal ageing” OR “productive aging” OR “productive ageing” OR “effective aging” OR “masticatory function” OR “masticatory functioning” OR “masticatory functions” OR mastication OR “well-being” OR wellbeing OR wellness OR “aesthetic appearance” OR “aesthetic appearances” OR “physical appearance” OR “physical appearances” OR “cognitive function” OR “cognitive functions” OR “cognitive functioning” OR “physical function” OR “physical functions” OR “physical functioning” OR “nutritional status” OR “nutrition status” OR “health outcome” OR “health outcomes” OR “health status” OR WHOQOL OR “WHO QOL” OR “healthy days measures”):ti,ab,kw

#8 [mh "Quality of Life"] OR [mh "Cognition"] OR [mh "Mastication"] OR [mh "Patient Satisfaction"] OR [mh "Healthy Aging"] OR [mh "Cognitive Aging"] OR [mh "Health Status"] OR [mh "Nutritional Status"] OR [mh "Physical Appearance, Body"]

#9 #7 OR #8

#10 #3 AND #6 AND #9
